# Higher programmed cell death 1 ligand 1 (PD-L1) mRNA level in clear cell renal cell carcinomas is associated with a favorable outcome due to the active immune responses in tumor tissues

**DOI:** 10.18632/oncotarget.13765

**Published:** 2016-12-01

**Authors:** Xiang-hui Ning, Yan-qing Gong, Shi-ming He, Teng Li, Jiang-yi Wang, Shuang-he Peng, Jin-chao Chen, Jia-yuan Liu, Nie-nie Qi, Ying-lu Guo, Kan Gong

**Affiliations:** ^1^ Department of Urology, Peking University First Hospital, Beijing 100034, P.R China; ^2^ Institute of Urology, Peking University, Beijing 100034, P.R China; ^3^ National Urological Cancer Center, Beijing 100034, P.R China

**Keywords:** renal cell carcinoma, programmed death 1 ligand-1, prognosis, immune response

## Abstract

Renal cell carcinoma is one of the most common urological tumors. The role of programmed cell death 1 ligand 1 (PD-L1) in renal cell carcinomas in predicting outcome of the patients is yet unclear. We analyzed the clinical and RNA-seq data of 522 kidney clear cell cancer, 259 kidney papillary cell carcinoma and 66 kidney chromophobe patients from The Cancer Genome Atlas (TCGA) database. In kidney clear cell cancer patients with high PD-L1 mRNA level and low PD-L1 mRNA level in tumors, the median overall survival periods were 45.0 and 37.1 months respectively (p=0.002). Multivariate Cox regression tests found that PD-L1 mRNA level in tumor was an independent predictor for overall survival status in kidney clear cell cancer patients (HR=0.7, 95% CI 0.5-0.9, p=0.007). However, no significant difference in overall survival status was found between high and low PD-L1 groups in kidney papillary cell carcinoma and kidney chromophobe cohorts. Gene-set enrichment analysis on the data from databases of TCGA and GSE53757 dataset in Gene Expression Omnibus databases showed that several pathways relating to immunological functions were activated in kidney clear cell cancers with high PD-L1 mRNA expression, and glycolysis and epithelial-mesenchymal transition pathways relating to tumor progression and metastasis were increased in kidney clear cell cancers with low PD-L1 mRNA level. In conclusion, higher PD-L1 mRNA level in kidney clear cell cancer tissues was associated with a favorable outcome due to the higher immunological responses in tumor tissues.

## INTRODUCTION

Renal cell carcinoma (RCC) is estimated to be the ninth leading cause of cancers in the US [1]. Three subtypes taking up 95% cases of RCC are clear cell RCC (KIRC), kidney papillary carcinoma (KIRP) and kidney chromophobe (KICH) [2]. The five-year overall survival rate of RCC is about 74%. The prognosis of RCC patients is closely related to patients’ age, tumor grade, and TNM stage [3]. Recently, mutations in *PBRM1*, *BAP1* and *SETD2* are identified to be the molecular biomarkers for the prognosis of RCC [4, 5]. Beyond predicting prognosis, molecular biomarkers may also provide tumorigenic characteristics that are useful for the development of novel anti-RCC therapies [5].

Programmed cell death 1 ligand 1 (PD-L1, CD274, B7-H1) expressed on antigen presenting cells, B cells and other tissue cells can bind its receptor PD-1 on T cells, B cells and myeloid cells to negatively regulate immune responses [6]. In RCC patients, PD-L1 expressed on tumor cells detected by immunohistochemistry was considered to be a risk factor for prognosis, but other studies found that higher PD-L1 mRNA level in RCC tissues estimated by RNA-seq approach was recognized as an indicator of favorable prognosis [7–12]. Extensive studies are therefore required to compromise the contrary results.

The prognosis of locally advanced or metastatic RCC is poor. Targeting therapy directly inhibiting the specific molecules such as tyrosine kinase or mammalian target of rapamycin (mTOR) has better clinical responses than cytokine therapy, but many patients become refractory to these therapies after a period of the treatment [2]. Recently, checkpoint inhibitors targeting PD-1 or its ligand have been introduced and the clinical trial is ongoing [6, 13, 14]. Primary results indicate that clinical response rate to the checkpoint inhibitors ranges from 11.7-29% in RCC patients [6]. To improve the prognosis of advanced RCC patients, the optimized regimens of systemic therapies need to be explored.

In this study, we aimed to investigate the role of PD-L1 mRNA expression in tumors in predicting the outcome of RCC based on the analyses of the clinical and RNA-seq data presented in The Cancer Genome Atlas (TCGA) database. Gene-set enrichment analysis (GSEA) on the data in TCGA and Gene Expression Omnibus (GEO) databases contributes to comprehend the immunological changes in RCC and to provide potential strategies for systemic therapy of RCC.

## RESULTS

### Description of the integrated RCC data in TCGA

The integrated data of 522 KIRC, 259 KIRP and 66 KICH patients in TCGA were enrolled for analyses (Supplementary data 1). Demographic, clinical, follow-up and tumor pathological features of the three RCC subtypes are listed in Table 1. Among the three RCC subtypes, 174 (33.3%) KIRC patients, 41(15.8%) KIRP patients and 16 (24.2%) KICH patients died in the follow-up period (Table 1).

**Table 1:** Patient and tumor characteristics of the three RCC subtype cohorts in TCGA

Variable	KIRC	KIRP	KICH
Sample (n)	522	259	66
Median age (year)	61 (26-90)	62 (28-88)	50 (17-86)
Median PD-L1	40.8 (0-5361.1)	23.6 (0-640.6)	67.4 (0.5-2930.8)
Gender			
Male	337 (64.6%)	191 (73.7%)	39 (50.1%)
Female	185 (35.4%)	68 (26.3%)	27 (40.9%)
Laterality			
Left	248 (47.5%)	144 (55.6%)	30 (45.5%)
Right	273 (52.3%)	113 (43.6%)	36 (54.5%)
Others	1 (0.2%)	2 (0.8%)	
Clinical stage			
Stage I	260 (49.8%)	172 (66.4%)	21 (31.8%)
Stage II	56 (10.7%)	21 (8.1%)	25 (37.9%)
Stage III	123 (23.6%)	51 (19.7%)	14 (21.2%)
Stage IV	83 (15.9%)	15 (5.8%)	6 (9.1%)
Tumor stage			
T1	265 (50.8%)	175 (67.6%)	21 (31.8%)
T2	68 (13.0%)	24 (9.3%)	25 (37.9%)
T3	178 (34.1%)	56 (21.6%)	18 (27.3%)
T4	11 (2.1%)	2 (0.8%)	2 (3.0%)
Survival status			
Alive	348 (66.7%)	218 (84.2%)	50 (75.8%)
Died	174 (33.3%)	41 (15.8%)	16 (24.2%)

RCC subtype, KIRC: kidney clear cell carcinoma; KIRP: kidney papillary carcinoma; KICH: kidney chromophobe

PD-L1 mRNA levels of the three RCC subtype cohorts were extracted from the RNA-seq2 data, which displayed continuous variables with a wide range of 0 to 5,361.1. The three RCC subtype cohorts were further divided into high PD-L1 group and low PD-L1 group based on the median PD-L1 mRNA value (Table 1) [15, 16].

### PD-L1 mRNA level and survival status

**Figure 1:**
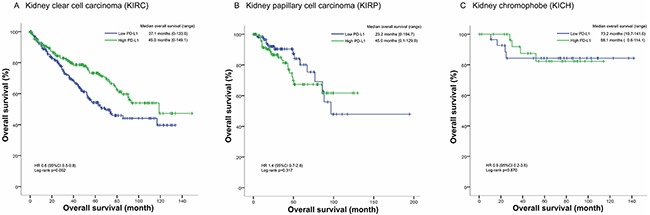
Kaplan-Meier plots of the KIRC, KIRP and KICH cohorts

In KIRC cohort, patients in high PD-L1 group had a median overall survival of 45.0 months (0-149.1 months) longer than the median overall survival of 37.1 months (0-133.6 months) in low PD-L1 group. The overall survival status is significantly different between high PD-L1 group and low PD-L1 group (HR=0.6, 95% CI 0.5-0.8, p=0.002; Figure 1). However, no significant difference in overall survival status was found between high PD-L1 group and low PD-L1 group in KIRP and KICH cohorts (Figure 1). Then we included the variables including age, gender, laterality, tumor grade, clinical stage, tumor stage, metastasis, and PD-L1 mRNA level into a multivariate Cox regression model and found that PD-L1 mRNA level was an independent predictor for overall survival status of KIRC patients (HR=0.7, 95% CI 0.5-0.9, p=0.007; Table 2).

**Table 2:** Univariate and multivariate regression analyses for predicting overall survival in KIRC cohort

Variable	Univariate		Multivariate	
	HR (95% CI)	p-value	HR (95% CI)	p-value
Age	1.8 (1.3-2.4)	<0.001*	1.6 (1.2-2.1)	0.003*
Gender	1.1 (0.8-1.4)	0.741	1.2 (0.9-1.6)	0.326
Laterality	0.7 (0.5-1.0)	0.024*	0.8 (0.6-1.0)	0.070
Tumor grade	2.2 (1.8-2.7)	<0.001*	1.5 (1.2-1.9)	<0.001*
Clinical stage	1.9 (1.6-2.1)	<0.001*	1.6 (1.4-1.9)	<0.001*
Tumor stage	1.9 (1.6-2.2)	<0.001*	0.8 (0.6-1.1)	0.109
Lymph node metastasis	0.9 (0.8-1.1)	0.267	0.9 (0.8-1.1)	0.187
Distant metastasis	2.3 (1.8-2.9)	<0.001*	1.1 (0.7-1.8)	0.643
PD-L1 mRNA level	0.6 (0.5-0.8)	0.002*	0.7 (0.5-0.9)	0.007*

* : statistically significant to predict overall survival rate

### PD-L1 mRNA level and clinical features of KIRC cohort

In addition to the significant difference in overall survival status between low PD-L1 group and high PD-L1 group, no differences were detected in clinical characteristics including age, laterality, clinical stage, tumor stage, metastasis, and tumor grade, except for a higher male ratio in low PD-L1 group (p=0.026, Table 3).

**Table 3:** Comparison of clinical characteristics between low PD-L1 group and high PD-L1 group in KIRC cohort

	Group		p-Value
	Low PD-L1	High PD-L1	
Sample (n)	261	261	
Age (year)			0.381
≤61	133 (51.0%)	143 (54.8%)	
>61	128 (49.0%)	118 (45.2%)	
Gender			0.022*
Male	181 (69.3%)	156 (59.8%)	
Female	80 (30.7%)	105 (40.2%)	
Laterality			0.601
Left	123 (47.1%)	125 (47.9%)	
Right	137 (52.5%)	136 (52.1%)	
Others	1 (0.4%)	0	
Clinical stage			0.082
Stage I	133 (50.9%)	127 (48.7%)	
Stage II	19 (7.3%)	37 (14.2%)	
Stage III	66 (25.3%)	57 (21.8%)	
Stage IV	43 (16.5%)	40 (15.3%)	
Tumor stage			0.056
T1	135 (51.7%)	130 (49.8%)	
T2	26 (10.0%)	42 (16.1%)	
T3	97 (37.2%)	81 (31.0%)	
T4	3 (1.1%)	8 (3.1%)	
Lymph node metastasis			0.208
N0	109 (41.8%)	129 (49.4%)	
N1	9 (3.4%)	7 (2.7%)	
NX	143 (54.8%)	125 (47.9%)	
Distant metastasis			0.620
M0	207 (79.3%)	214 (82.0%)	
M1	41 (15.7%)	38 (14.6%)	
MX	13 (5.0%)	9 (3.4%)	
Tumor grade			0.609
G1	6 (2.3%)	6 (2.3%)	
G2	104 (39.8%)	121 (46.4%)	
G3	110 (42.1%)	95 (36.4%)	
G4	39 (15.0%)	36 (13.8%)	
GX	2 (0.8%)	3 (1.1%)	
Survival status			0.002*
Alive	157 (60.2%)	191 (73.2%)	
Died	104 (39.8%)	70 (26.8%)	

* : statistically significant

### Gene expression signature in high PD-L1 group and low PD-L1 group of KIRC cohort

**Figure 2:**
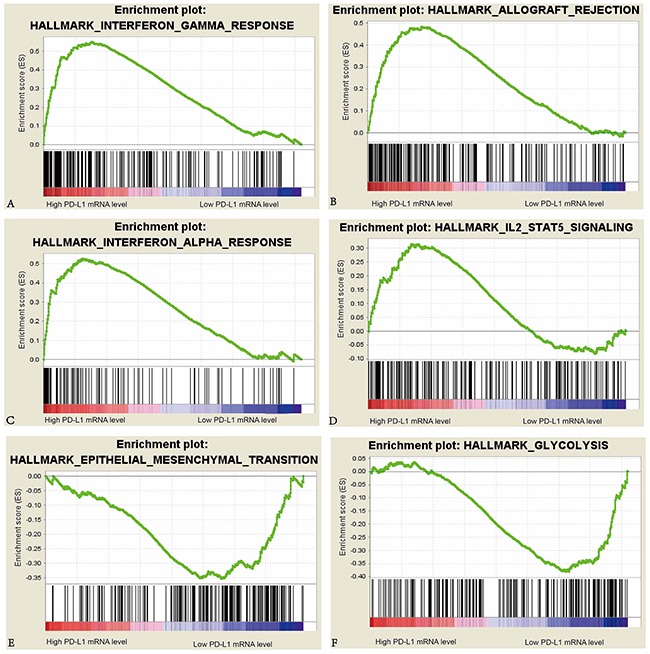
Enrichment plots of interferon-γ response, interferon-α response, epithelial mesenchymal transition, allograft rejection, IL2 Stat5 signaling, and glycolysis against PD-L1 mRNA level in the KIRC cohort

We further analyzed the gene expression data in tumors to compare the differences in cell processes such as immune, proliferation, metabolism and DNA damage repair between high PD-L1 group and low PD-L1 group in the KIRC cohort (Table 4). We also performed the same analyses for the 72 KIRC cases in GSE53757 dataset of GEO database to confirm the differences of cell processes between the two groups in KIRC cohort. A total of 10 pathways were upregulated in high PD-L1 group, and a total of 3 pathways were upregulated in low PD-L1 group of KIRC patients in both TCGA and GEO databases. In high PD-L1 group, at least 8 of the 10 upregulated pathways are closely related to immunological functions. In contrast in low PD-L1 group, the 3 upregulated pathways are involved in tumor progression and metastasis (Figure 2).

**Table 4:** Pathway analyses for high PD-L1 group and low PD-L1 group in KIRC cohort from TCGA and GEO databases

	KIRC from TCGA (522 cases)				KIRC from GSEA 53757 in GEO (72 cases)			
	High PD-L1	q-val.	Low PD-L1	q-val.	High PD-L1	q-val.	Low PD-L1	q-val.
1	Interferon-γ response*	<0.001	DNA repair	<0.001	Allograft rejection*	<0.001	Epithelial mesenchymal transition*	<0.001
2	Allograft rejection*	<0.001	MYC targets v2	<0.001	Interferon-γ response*	<0.001	Uv response down	<0.001
3	Interferon-α response*	<0.001	Myogenesis*	0.001	Interferon-α response*	<0.001	Angiogenesis	<0.001
4	Protein secretion	<0.001	MYC targets v1	0.004	IL6 JAK Stat3 signaling*	<0.001	Myogenesis*	<0.001
5	Mitotic spindle	<0.001	Glycolysis	0.003	E2F targets	<0.001	TGF–β signaling	<0.001
6	Inflammatory response*	<0.001	Epithelial mesenchymal transition*	0.008	Inflammatory response*	<0.001	Hypoxia	<0.001
7	G2M checkpoint*	0.001	Coagulation*	0.041	G2M checkpoint*	<0.001	Notch signaling	0.002
8	Androgen response	0.002	Oxidative phosphorylation	0.043	TNF-α signaling via NF-κB*	<0.001	Apical junction	<0.001
9	IL6 JAK Stat3 signaling*	0.006			Complement*	<0.001	Wnt β-catenin signaling	0.004
10	Kras signaling up	0.005			IL2 STAT5 signaling*	<0.001	Hedgehog signaling	0.018
11	Complement*	0.010			PI3K AKT mTOR signaling*	0.004	Androgen response	0.008
12	TNF-α signaling via NF-κB*	0.010					Coagulation*	0.002
13	Uv response down	0.014					Bile acid metabolism	0.002
14	IL2 Stat5 signaling*	0.018					Fatty acid metabolism	0.004
15	PI3K AKT mTOR signaling*	0.019					Adipogenesis	0.004
16							Xenobiotic metabolism	0.006
17							Kras signaling up	0.008
18							Estrogen response early	0.012

* : upregulated both in KIRC patients in TCGA and GEO databases; q-val.: FDR q-value

## DISCUSSION

In this study, we identified that PD-L1 mRNA level in tumor tissue was an independent prognosis predictor for KIRC patients and that the activation of functional pathways was different in KIRCs with different PD-L1 mRNA levels.

Previous studies using immunohistochemistry and ELISA to measure PD-L1 protein in tumors and sera reached the conclusion that higher PD-L1 level was associated with poor prognosis of the three subtypes of RCC [7, 8, 17]. Quantification of PD-L1 through the intensity of immunohistochemistry staining by different antibodies may bring ambiguous results [17], and may only represent the PD-L1 expression level in tumor cells. In our present study, we obtained the data of the three main subtypes of RCC from TCGA and processed by the same method. The results revealed that PD-L1 was an independent prognosis predictor for KIRC patients but not for KIRP and KICH patients. Recently, Messai et al. reported that mutations in von Hippel-Lindau (*VHL*) gene positively correlated with PD-L1 expression in KIRC cells but not in KIRP and KICH cells [18], suggesting that PD-L1 may play different role in different RCC subtypes and that anti-PD-1/PD-L1 therapy may not be suitable for all RCC patients.

It seems paradoxical that higher expression of immunosuppressive PD-L1 correlated with improved outcomes. This will be resolved if PD-L1 expression is viewed as a reflection of the presence of endogenous antitumor immunity [19]. In other words, higher PD-L1 mRNA level in tumors is the negative feedback to the activated antitumor responses such as IFN-γ response, IFN-α response and activated IL2-Stat5 signaling pathway in tumor microenvironment [19]. The outcome of a tumor is determined by the interaction between host antitumor immune responses and negative feedback to the immunological responses in tumor [19]. In the KIRC cohort, patients with active immune responses usually had higher PD-L1 mRNA level in tumors and better outcomes, while those with less active immune responses and increased glycolysis and epithelial-mesenchymal transition had lower PD-L1 mRNA level in tumors and shorter survival periods.

Treatment strategies for tumors with different status of endogenous immune responses should be different [20]. Recently, a prospective study revealed that tumors with higher PD-L1 expression had a better response to high-dose IL-2 than those with negative PD-L1 expression [21]. The tumors with active antitumor immune responses indicate that both innate and adaptive immune responses are strongly activated to eliminate tumor cells with specific antigens on their surfaces [22]. In view of the better response to high-dose IL-2 and the immunosuppressive effect of higher PD-L1 [21], the treatment of KIRC with higher PD-L1 expression should combine the therapies promoting host antitumor immune responses such as IL-2 and blocking the immunosuppressive status such as anti-PD-L1/PD-1 antibody therapies. In contrast in KIRC with lower response to high-dose IL-2 and lower PD-L1 mRNA expression, the weak immune response may attribute to the lack of tumor-specific antigens on tumor cells and the secretion of immunosuppressive cytokines such as VEGF and TGF-β [22]. Molecular target therapy may be useful for these patients. PD-L1 mRNA level in KIRC may be used as a reference for drug treatment strategies of KIRC patients. However, the different treatment regimen we propose for KIRC with different PD-L1 mRNA level in tumors must be tested further by random clinical trials. One limitation of this study is the lack of another independent cohort for validation. In addition, other factors which can also influence the outcome of KIRC patients are not taken into account due to the lack of the data, such as the time of disease recurrence after surgery and the treatment for the patients.

In conclusion, our study provides a new insight into the significance of PD-L1 in KIRC. Higher PD-L1 mRNA level was associated with a better outcome of the patients. The underlying mechanism may be the higher antitumor immune responses in the microenvironment of KIRC. PD-L1 mRNA level in tumor may be one of the factors affecting the outcome of KIRC patients, and may also be a reference for drug treatment strategy for these patients.

## MATERIALS AND METHODS

### Patients and data collection

Clinical, follow-up and RNA-seq data of the 536 KIRC, 291 KIRP and 66 KICH patients were obtained from TCGA by cBioportal platform and TCGA-Assembler [23, 24]. The patients with integrated clinical stage, T stage, overall survival information and mRNA levels in tumor were enrolled in this study. mRNA expression profiling by array of the 72 KIRC tumors in GSE53757 dataset in GEO were also included [25]. The data used in this study are opened to public for access without limitation and restriction. This study was performed according to the publication guidelines provided by TCGA ( http://cancergenome.nih.gov/publications/publicationguidelines).

### Pathway analysis

Gene-set enrichment analysis (GSEA) was used to identify the pathways in two different PD-L1 mRNA level groups [26]. RNA-seq data were processed by TCGA-Assembler, and a total of 20,486 genes were enrolled for GSEA analyses. In addition, 20,282 genes from GSE53757 dataset were used for validating the pathway analyses. In the processes of GSEA analyses, the hallmark gene sets (h.all.v5.1.symbols.gmt) were used [27]. The p value of GSEA was computed by 1,000-gene-set two-sided permutation test.

### Statistical methods

KIRC, KIRP and KICH patients were divided into two groups according to the median value of PD-L1 mRNA level in tumors. Comparisons of demographic, clinical and pathological features between the two PD-L1 mRNA level groups were conducted using chi-square test or Fisher exact test. Overall survival was assessed using Kaplan-Meier method and log-rank test. Hazard ratio (HR) was calculated by Cox regression model and the result was provided as HR value and 95% confidence interval of the HR. In order to investigate whether PD-L1 level was an independent predictor for outcome in KIRC cohort, we included all the variables in multivariate Cox regression test using a backward conditional approach and eliminated the variables that the p value was >0.05. FDR q value was used for the evaluation of different pathways in different groups. Statistical tests were analyzed using SPSS 19.0 software (SPSS, Inc., Chicago, IL, USA). The p value of <0.05 was considered to be statistically significant.

## SUPPLEMENTARY MATERIALS DATA




