# Supplementary material for: Higher programmed cell death 1 ligand 1 (PD-L1) mRNA level in clear cell renal cell carcinomas is associated with a favorable outcome due to the active immune responses in tumor tissues
Source: Oncotarget. 2016 Dec 1;8(2):3355–63. doi: 10.18632/oncotarget.13765 (PMC5356887; doi:10.18632/oncotarget.13765)
Supplement: Supplementary file 1 [file oncotarget-08-3355-s001.pdf]

Higher programmed cell death 1 ligand 1 (PD-L1) mRNA level in clear cell renal cell carcinomas is associated with a favorable outcome due to the active immune responses in tumor tissues

Supplementary Data

Supplementary Data1: Patient ID number in TCGA database.

See Supplementary File 1
